# Multiple myeloma and fever of unknown origin: a need for therapy

**DOI:** 10.1038/sj.bjc.6602693

**Published:** 2005-07-12

**Authors:** N Mumoli, M Cei

**Affiliations:** 1 Department of Internal Medicine, Livorno Hospital, Viale Alfieri 36, 57100 Livorno, Italy

**Sir**,

In their concise review on evolving treatment strategies for myeloma, Morgan and Davies (2005) stated that ‘early death was, and still is, a significant problem, and addressing the causes of this is important including early diagnosis, the treatment of infection, hydration and the choice of the most appropriate chemotherapy’. The most common presenting symptoms of the disease are fatigue, bone pain and recurrent infections. The latest classification system, recently released from The International Myeloma Working Group (2003), highlighted the importance to distinguish between asymptomatic and overt myeloma, because only the latter requires active treatment. According to this statement, the biological activity of the disease, more than the tumour mass, is the foremost guide to the therapy. The related organ or tissue impairment (ROTI) that meets the criterion for the diagnosis consists of hypercalcemia, renal insufficiency, anaemia or bone lesions (a group of findings referred to as CRAB). However, we need to understand that some of the clinical features of the disease, as the degree of anaemia, are not completely explained by the plasma cellular infiltrates or paraprotein secretion. Although uncommonly, myeloma can present as fever of unknown origin (FUO) (Mueller *et al*, 2002; Lambotte *et al*, 2003). In these cases the fever is attributable to the disease itself, and indeed no infections are demonstrable, even with extensive work-up. Moreover, the fever cannot be abated with antibiotics although chemotherapy works well (Mumoli *et al*, 2004). It is not already known if the modulation of the interaction between the neoplastic clone and the marrow microenvironment, as is the case during therapy with bortezomib, could reverse this kind of presentation; this could in turn result in delaying chemotherapy, at least in patients not eligible for transplantation procedures. Morgan and Davies conclude saying that in the future it will be desirable to describe myeloma as a treatable disease with prolonged survival and good quality of life if the correct treatment decisions are made. We agree with this and we add, moreover, if the correct diagnosis has also been made. We therefore suggest that FUO should be mentioned as an indication for diagnosis and therapy in myeloma patients.
